# Hepcidin deficiency and iron deficiency do not alter tuberculosis susceptibility in a murine *M*.*tb* infection model

**DOI:** 10.1371/journal.pone.0191038

**Published:** 2018-01-11

**Authors:** Rachel Harrington-Kandt, Elena Stylianou, Lucy A. Eddowes, Pei Jin Lim, Lisa Stockdale, Nawamin Pinpathomrat, Naomi Bull, Janet Pasricha, Marta Ulaszewska, Yulia Beglov, Sophie Vaulont, Hal Drakesmith, Helen McShane

**Affiliations:** 1 Jenner Institute, University of Oxford, Oxford, United Kingdom; 2 MRC Human Immunology Unit, Weatherall Institute of Molecular Medicine, University of Oxford, Oxford, United Kingdom; 3 Department of Immunology and Infection, London School of Hygiene & Tropical Medicine, London, United Kingdom; 4 Wellcome Trust Centre for Human Genetics, University of Oxford, Oxford, United Kingdom; 5 Institut Cochin, INSERM 567, CNRS 8104, Université Paris 5, Paris, France; Lady Davis Institute for Medical Research, CANADA

## Abstract

Tuberculosis (TB), caused by the macrophage-tropic pathogen *Mycobacterium tuberculosis (M*.*tb)* is a highly prevalent infectious disease. Since an immune correlate of protection or effective vaccine have yet to be found, continued research into host-pathogen interactions is important. Previous literature reports links between host iron status and disease outcome for many infections, including TB. For some extracellular bacteria, the iron regulatory hormone hepcidin is essential for protection against infection. Here, we investigated hepcidin (encoded by *Hamp1*) in the context of murine *M*.*tb* infection. Female C57BL/6 mice were infected with *M*.*tb* Erdman via aerosol. Hepatic expression of iron-responsive genes was measured by qRT-PCR and bacterial burden determined in organ homogenates. We found that hepatic *Hamp1* mRNA levels decreased post-infection, and correlated with a marker of BMP/SMAD signalling pathways. Next, we tested the effect of *Hamp1* deletion, and low iron diets, on *M*.*tb* infection. *Hamp1* knockout mice did not have a significantly altered *M*.*tb* mycobacterial load in either the lungs or spleen. Up to 10 weeks of dietary iron restriction did not robustly affect disease outcome despite causing iron deficiency anaemia. Taken together, our data indicate that unlike with many other infections, hepcidin is decreased following *M*.*tb* infection, and show that hepcidin ablation does not influence *M*.*tb* growth *in vivo*. Furthermore, because even severe iron deficiency did not affect *M*.*tb* mycobacterial load, we suggest that the mechanisms *M*.*tb* uses to scavenge iron from the host must be extremely efficient, and may therefore represent potential targets for drugs and vaccines.

## Introduction

Iron is an essential element required by almost all organisms, due to its role in a range of key cellular processes such as DNA synthesis and respiration. During infection, iron availability to both host and pathogen may impact on infectious outcome. Since both the host and the pathogen require iron, the pathogen must be able to scavenge iron from the host, and the host alters its iron distribution in response to infection. This redistribution may serve as a protective mechanism against some pathogens, such as the extracellular bacterium *Vibrio vulnificus* [1], which is termed siderophilic because excess iron enhances its pathogenicity. Host iron status impacts on the severity of many infectious diseases including TB [2, 3], HIV [4], and malaria [5].

In humans, the majority of iron in circulation is derived from the recycling of senescent red blood cells by macrophages and a relatively small amount is obtained daily through the absorption of dietary iron [6]. Since excretion of iron is not modulated, iron storage and release must be tightly regulated in order to prevent iron deficiency, iron toxicity, and to prevent excess iron availability for pathogens. Hepcidin, a 25 amino acid peptide hormone produced in the liver, is a major regulator of iron homeostasis [7]. Hepcidin regulates systemic iron levels as well as the redistribution of tissue iron by binding to the iron exporter ferroportin, causing it to be degraded [8]. Ferroportin is highly expressed on macrophages and enterocytes [9]. Hepcidin decreases serum iron concentrations by redistributing iron into macrophages, and impairing dietary iron absorption. As a result, excess hepcidin can cause anaemia since iron availability for erythropoiesis is decreased. On the other hand, low hepcidin leads to excessively high systemic iron levels as a result of increased dietary iron uptake and iron release from erythrophagocytic macrophages. Hereditary haemochromatosis is an iron overload disorder resulting from genetic mutations leading to defective hepcidin production [10, 11]. Since hepcidin regulates the distribution of iron in the body, it has variable impacts on the outcome of infection, depending on the niche of the pathogen [12, 13]. For example, high hepcidin decreases susceptibility to some blood-borne bacteria [1] and can also protect against liver stage *Plasmodium* infection [5]. Macrophage iron is increased when hepcidin is high, and this could result in increased replication of macrophage-tropic pathogens. Therefore, changes in hepcidin and iron homeostasis are likely to have entirely different effects on pathogenicity depending on the nature of the pathogen.

The regulation of hepcidin is complex; a number of factors including iron, erythroid drive, inflammation and hypoxia are involved [12]. Increased systemic iron induces hepcidin via the bone morphogenetic protein (BMP)/SMAD signalling pathway, of which Bmp6 is an essential regulator [14, 15]. Hepcidin is also induced by a wide range of infectious stimuli in mice, for example *Vibrio vulnificus* [1], Influenza A virus [13], *Candida albicans* [13], *Plasmodium* species [5], and *Salmonella typhimurium* [16]. During infection, inflammation can induce hepcidin through the interleukin 6 (IL6)/ signal transducer and activator of transcription 3 (STAT3) pathway. Persistent upregulation of hepcidin can lead to anaemia of chronic disease (ACD) [17, 18]. Conversely, anaemia and erythroid drive inhibit hepcidin production at least in part through erythroferrone [19].

Tuberculosis (TB) is an airborne infection, transmitted through the inhalation of *M*.*tb* infected droplets into the lungs, where it is phagocytosed by resident alveolar macrophages [20, 21]. It is well documented that *M*.*tb* requires iron in order to survive, and iron and haemoglobin have been associated with BCG growth *in vitro* [22], but little is known about the role of hepcidin in *M*.*tb* infection *in vivo*. Recent studies in humans highlight an associative relationship between high hepcidin and increased susceptibility to TB, although a number of these studies may be confounded by HIV co-infection [23–25]. In addition, high hepcidin and ferritin have also been associated with increased risk of progression to TB disease in close household contacts of *M*.*tb* infected individuals [26]. In mice, iron overload in beta-2 microglobulin knockout animals has been associated with increased susceptibility to *M*.*tb* infection [27]. However, more recently neither hepcidin knockout mice, nor wild-type mice with increased iron levels were shown to have increased susceptibility to *M*.*tb* [28].

In this study, we investigated hepcidin and iron homeostasis in a murine *M*.*tb* infection model. We show hepcidin expression decreases during murine *M*.*tb* infection, confirm that hepcidin knockout mice are not more susceptible to *M*.*tb* infection and furthermore find that even severe iron deficiency does not alter *M*.*tb* burden.

## Methods

### Animals

Animals used in time course and iron restriction studies were female C57BL/6 mice, purchased from Envigo, UK, or female BALB/c mice (Harlan Laboratories, UK). Animals were age matched for experiments. Procedures were performed in accordance with the UK Home Office regulations (Scientific Procedures Act 1986) under project license 30/2889 granted by the UK Home Office. *Hamp1*^*-/-*^ mice on a C57BL/6 J129 background (backcrossed onto C57BL/6 for at least 10 generations) were generated as described previously [29]. Wild type littermate mice were used as controls. Only females were used for experiments. Animals were sacrificed humanely at the end of each experiment.

### Custom diets

For all experiments with the exception of the iron restricted diet experiments, mice were fed normal chow and water *ad libitum*. Custom research diets were produced by Envigo, UK and were irradiated and vacuum packed. The iron deficient diet (TD. 99397) contained 2–6 parts per million (ppm) of iron in the form of ferric citrate. Controls for this experiment were fed an otherwise identical diet with the exception of an iron content of 200 ppm (TD.07801), a similar amount of iron as the standard diet in our facility. Animals were fed diet for the duration indicated in each study outline.

### *M*.*tb* aerosol infection

Animals were exposed to aerosolised *M*.*tb* Erdman KO1 (TMC107) (BEI Resources, Manassas, USA) using a Biaera AeroMP-controlled nebuliser (Biaera technologies, Hagerstown, USA) contained within a Biosafety level 3 TCOL isolator (Total Containment Oxford Limited, Oxford, UK) maintained under negative pressure with respect to the atmosphere. Mice were loaded into nose-only restrainers and exposed to *M*.*tb* for 10 minutes, followed by a 5 minute purge cycle with air flow at 12L/min and a pressure of 20psig (1.4x10^5^ Pa). For the low dose exposure (50–100 CFU), *M*.*tb* was prepared at 1x10^6^ CFU/ml and for high dose experiments (100–200 CFU), *M*.*tb* was prepared at 5x10^6^/ml in the nebuliser. Target infectious dose was verified by quantifying CFU in 2 mice 24 hours post-infection. All work on infected tissue was performed at Biosafety Level (BSL) 3 within a Class I biological safety cabinet according to standard protocol in full compliance with all national and local health and safety regulations.

### Quantification of CFU

Four weeks post-infection, lungs and spleens of infected animals were aseptically removed and homogenised in 1ml of phosphate-buffered saline (PBS) in reinforced homogenising tubes containing 2.8mm ceramic beads (Stretton Scientific, UK). Organs were homogenised in a Precellys® 24 homogeniser (Stretton Scientific, UK). Organ homogenates were serially diluted in PBS and plated on Middlebrook 7H11 (BD) plates containing OADC (Becton Dickinson) and glycerol (Sigma-Aldrich), following the manufacturers recommendations. Plates were incubated 37°C and CFU was enumerated 3 weeks later.

### RNA extraction

The median lobe of the liver was extracted and either snap-frozen in liquid nitrogen, or stored in RNA*later* (Qiagen) until required. RNA was extracted using an RNEasy Plus Mini Kit (Qiagen) and reverse transcribed using the High Capacity cDNA Reverse Transcription Kit (Applied Biosystems) following the manufacturer’s recommendations.

### Quantitative real-time PCR (qRT-PCR)

Gene expression was quantified by qRT-PCR using TaqMan Gene Expression Mastermix and TaqMan Gene Expression assays (S1 Table), following the manufacturer’s protocol. qRT-PCR was performed on the Applied Biosystems 6500 Fast Real-Time PCR system machine. Ct values were obtained by setting dye fluorescence threshold at 0.2. Changes in gene expression was quantified relative to the endogenous control *Hprt1* (encoding hypoxanthine-guanine phosphoribosyltransferase) using the 2^dCt method analysis, where dCt is the difference in Ct values between Hprt1 and gene of interest. Ct values for *Hprt1* were not significantly different between iron deficient and iron replete groups of mice.

### Genotyping of Hamp1^-/-^ mice and wild type littermate controls

Genomic DNA was extracted from ear punch biopsies by incubating the biopsies in 20μl of ear punch buffer containing 1mg/ml proteinase K (Qiagen), 50mM Tris pH 8, 10mM EDTA, 1% SDS and 2mM NaCl in dH_2_O for 20 minutes at 55°C, followed by a vortex and a further incubation at 20 minutes at 55°C. 180μl of dH_2_O was then added and tubes were heated at 99°C for a further 5 minutes in order to inactivate the proteinase K.

PCR was performed using a GoTaq Flexi DNA polymerase kit (Promega) and dNTPs from a High Capacity RNA-to-cDNA kit (Applied Biosystems), following the manufacturer’s recommendations. Separate wild type and knockout reactions were performed using the same reverse primer. Custom-made lyophilized oligonucleotides (Sigma Aldrich) (forward wild type: 5’-GGG CTG TAG AGG TTC TGC TG-3’, Reverse: 5’- AAC AGA TAC CAC ACT GGG AA-3’, Forward knockout: 5’- GCC TGA AGA ACG AGA TCA GC-3’) were diluted to a final concentration of 160nM per reaction. Wild type reactions amplify a 586 base pair (bp) product and knock out reactions amplify a 260bp product.

### Blood drawing for haematological analyses

Mice were terminally anaesthetised with 2–4% isofluorane and oxygen (2L/min) in air and blood drawn via cardiac puncture using a BD Microlance 3 0.4mmx16mm needle with BD Plastipak 1ml syringe into a BD Microtainer tube containing di-potassium EDTA. Assessment of haematological parameters was performed using a Pentra ABX or Sysmex KX21N machine.

### Tissue preparation and Perl’s staining

Mice were culled by cervical dislocation. The spleen and the median lobe of the liver were aseptically removed and transferred to a 50ml falcon tube containing 10ml of 10% Neutral Buffered Formal (NBF). To harvest lungs, the body cavity was opened up to expose the trachea. A nick was then created in the top of the trachea using a scalpel. A BAL tube attached to a 2ml syringe containing 1ml of 10% NBF was then gently inserted into the trachea and the lungs were then flushed with NBF. Lungs were then removed and placed in 10% NBF. Tissue was fixed overnight in 10% NBF. Samples were then transferred to 70% EtOH to start the dehydration process. Paraffin-embedded tissue sectioning and Perl’s staining (for non-haem iron) were performed by the Oxford Centre for Histopathology Research (OCHRe) (JR Hospital, Oxford).

### Quantification of tissue non-heme iron

Tissues were dried for 2 hours at 95°C before weighing, followed by digestion in 1ml of 10% (w/v) trichloroacetic acid/ 30% hydrochloric acid mix at 65°C for 20 hours. Samples were then cooled and reacted with working chromogen reagent at a ratio of 5:5:1 (distilled water: saturated (~75%w/v) sodium acetate: chromogen reagent (0.1% (w/v) bathophenanthrolinedisulfonic acid (BPS, 146617, Sigma)/0.8% thioglycolic acid (88652, Sigma). A standard curve was generated using a dilution series of ferric ammonium citrate (F5879, Sigma) in the 10% (w/v) trichloroacetic acid/ 30% hydrochloric acid mixture. Tissue non-heme iron content was calculated colorimetrically against the standard curve, at an OD of 535nm.

### Statistical analyses

All statistical analyses were performed using GraphPad Prism 6 software. Data sets were tested for normality using Kolmogorov Smirnov, Shapiro-Wilk and D’Agostino-Pearson tests. In the time course experiments, where 2 or more groups are compared, Kruskall-Wallis tests were performed with Dunn’s post-hoc test for multiple comparisons. All other comparisons between groups were Mann-Whitney tests, adjusted for multiple comparisons where necessary. Correlations were Spearman’s Rank Correlations.

## Results

### Effect of *M*.*tb* infection on the expression of hepcidin and other genes involved in iron homeostasis

**Fig 1:**
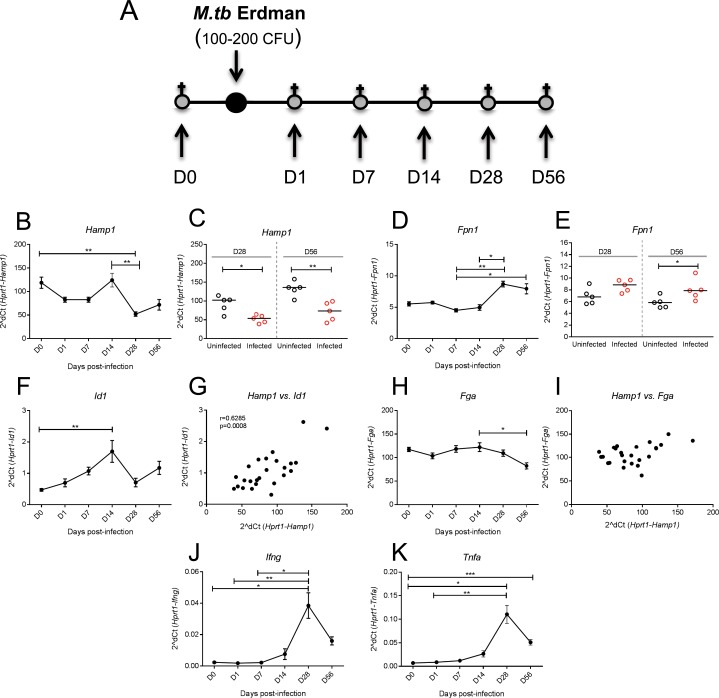
The effect of *M*.*tb* infection on the expression of genes involved in the regulation of iron homeostasis. Female 6–10 week old C57BL/6 mice were infected with 100–200 CFU of *M*.*tb* Erdman via aerosol. Animals were sacrificed at day (D) 0 (baseline), D1, D7, D14, D28 and D56 post-infection (outlined in **A**). Hepatic gene expression analyses over the time course are shown for *Hamp1* (**B**), *Fpn1* (**D**), *Fga* (**F**) and *Id1* (**H**). Comparisons of *Hamp1* and *Fpn1* gene expression respective to uninfected controls are depicted in **C** and **E** respectively. Correlations between *Hamp1* and *Id1*, and *Hamp1* and *Fga* are shown in **G** and **I**, respectively. Expression of immune genes *Ifng* and *Tnfa* are shown in figures **J** and **K,** respectively. Kruskal-Wallis tests with Dunn’s post-tests for multiple comparisons were done for time course studies, and Mann-Whitney tests for comparisons to uninfected controls. Correlations were Spearman’s rank correlations. In all cases *, **, *** and **** indicate p = <0.05, p = <0.01, p = <0.001 and p = <0.0001, respectively. Baseline values (D0) were not included in correlations. In panels C and E, black symbols represent uninfected animals and red symbols infected animals. In all other panels, animals are infected. N = 5 per group.

The iron regulatory hormone hepcidin is also an acute-phase response gene that is up-regulated rapidly in infection by the action of inflammatory cytokines particularly IL-6 [13]; chronic inflammation maintains hepcidin at a high level. *M*.*tb* is a relatively slow-growing pathogen associated with inflammation [30, 31] but how this infection influences hepcidin expression *in vivo* is not well understood. To address the effect of *M*.*tb* infection on the expression of hepcidin and other genes involved in iron homeostasis, we infected 6–10 week old female C57BL/6 mice with aerosolised *M*.*tb* and quantified hepatic gene expression at various time points post-infection between day 0 (before infection) to 56 days post-infection (D56) (outlined in Fig 1A). We found that the gene encoding hepcidin, *Hamp1*, fluctuated post-infection and was significantly reduced in infected animals by day 28 and day 56 post-infection (p = <0.05 and p = <0.01, respectively), in comparison to uninfected controls (Fig 1B and 1C); this decrease in hepatic *Hamp1* mRNA in infected versus uninfected animals was also observed 28 days after BALB/c mice were challenged with aerosolised *M*.*tb* (S1 Fig). Notably, there was no increase in *Hamp1* expression at earlier timepoints of infection. Hepatic expression of the gene encoding ferroportin (*Fpn1)* was up-regulated when compared with uninfected animals (day 56, p = <0.05, Fig 1D and 1E). To investigate whether Smad or Stat3 pathways may be involved in the observed down-regulation of *Hamp1* mRNA, we looked at the induction of genes induced by these two pathways. The pattern of expression over time of *Id1*, a Bmp pathway target gene, was similar to (Fig 1F) and correlated positively with hepcidin (r = 0.63, p = <0.001, Spearman’s correlation, Fig 1G). Conversely, the Stat3 target gene *Fga*, which encodes fibrinogen alpha did not correlate strongly with hepcidin (Fig 1H and 1I, r = 0.37, p = 0.066, Spearman’s correlation). Hepatic expression of *Ifng* and *Tnfa* increased as infection progressed, consistent with development of Th1 type immunity (Fig 1J and 1K).

### Effect of *Hamp1* deletion in mice on susceptibility to *M*.*tb*

**Fig 2:**
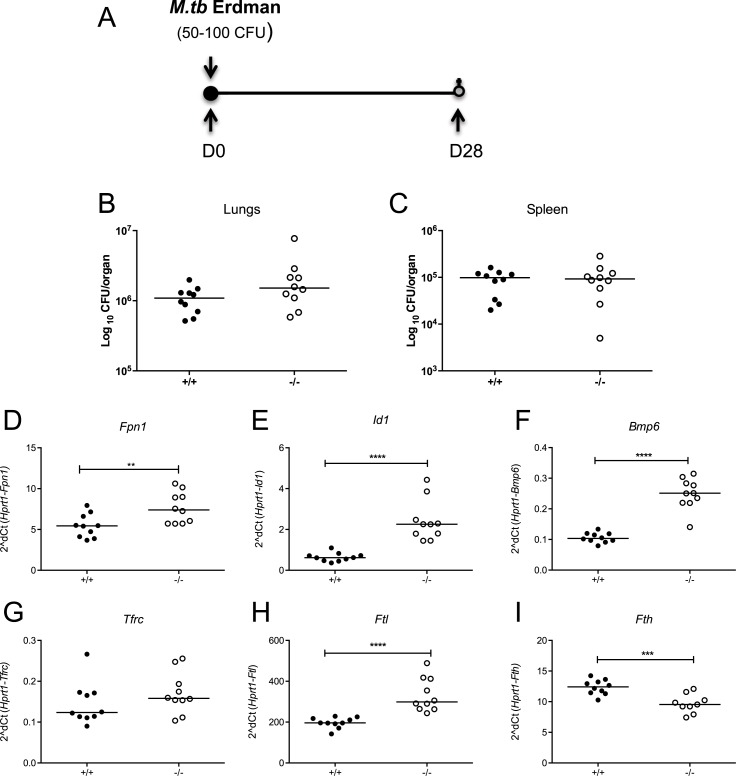
The effect of *Hamp1* deletion in murine *M*.*tb* infection and iron-related hepatic gene expression. Female 16–20 week old *Hamp1*^*-/-*^ mice and wild type controls were infected with 50–100 CFU of *M*.*tb* Erdman strain via aerosol (A). Animals were sacrificed 4 weeks later and CFU enumerated in organ homogenates (B-C). Hepatic gene expression is shown for *Fpn1* (D), *Id1* (E), *Bmp6* (F), *Tfrc* (G), *Ftl* (H) and *Fth* (I). Mann-Whitney tests were performed to compare groups where *, **, ***, and **** indicate p = <0.05, p = <0.01, p = <0.001 and p = <0.0001, respectively. N = 10 animals per group. Closed circles represent wild type animals and open circles represent *Hamp1*^*-/-*^ animals.

*Hamp1*-deficient mice have an iron-overloaded phenotype, with significant iron deposition in the lung and liver, sparing in the spleen, as well as increases in serum iron parameters and haemoglobin (S2 Fig), consistent with published reports [29]. We hypothesised that the observed down-regulation of *Hamp1* post-infection seen in our preliminary experiments could be a protective mechanism against *M*.*tb* infection and therefore that *Hamp1* deficiency might be beneficial in the context of *M*.*tb* infection. In order to investigate this, we infected female 16–20 week old mice lacking hepcidin with a low dose of *M*.*tb* (50–100 CFU) (Fig 2A). The median CFU in the lungs in the *Hamp1*^*-/-*^ animals was 1.52x10^6^ log_10_ CFU, not significantly different to 1.09x10^6^ log_10_ CFU in the wild type controls (p = 0.11, Fig 2B). Moreover, the median CFU burdens in the spleens were very similar (9.83x10^5^ log_10_ CFU in the *Hamp1*^*-/-*^ animals and 9.25x10^5^ log_10_ CFU in the wildtype controls, p = 0.99, Fig 2C). Therefore, *Hamp1* deletion did not significantly affect infectious outcome. In terms of gene expression, *Hamp1*^*-/-*^ mice had significantly higher hepatic levels of *Fpn1*, *Id1* and *Bmp6* mRNA compared with wildtype mice (p = <0.01, p = <0.0001, p = <0.0001 respectively, Fig 2D–2F), consistent with liver iron accumulation. We also measured hepatic mRNA levels of iron uptake and storage genes: expression of transferrin receptor (Tfrc) was not different between wild-type and *Hamp1* KO mice (Fig 2G), while *Hamp1*^*-/-*^ mice had significantly higher light chain ferritin mRNA (*Ftl)* than the controls, but significantly reduced heavy chain ferritin mRNA (*Fth) (*p = <0.0001 and p = <0.001, respectively, Fig 2H and 2I).

### The effect of dietary iron deficiency on the expression of genes involved in iron homeostasis

**Fig 3:**
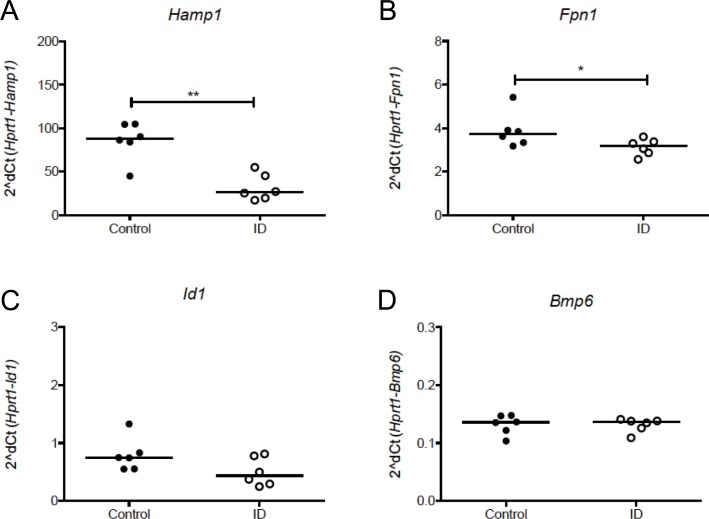
Characterisation of the iron deficient mouse model. Female 6–10 week old C57BL/6 mice were fed an iron deficient (2–6ppm) or control (200ppm) diet for a total of 2 weeks. Animals were sacrificed and hepatic gene expression analyses are shown for *Hamp1* (A), *Fpn* (B), *Id1* (C), and *Bmp6* (D). Mann-Whitney tests were performed to compare groups where *, **, *** and **** indicate p = <0.05, p = <0.01, p = <0.001 and p = <0.0001 respectively. N = 6 animals per group. Closed circles represent control animals and open circles represent iron deficient animals.

We next wished to investigate whether iron deficiency would influence *M*.*tb* infection. Initially, we characterised the effect of feeding female 6–10 week old mice an iron deficient or control diet *ad libitum* for a total of 2 weeks. Hepatic *Hamp1* mRNA was significantly reduced in mice fed low iron diet compared with controls (p = <0.01, Fig 3A). Hepatic expression of *Fpn1* was also significantly reduced in animals fed the iron deficient diet (p = <0.05, Fig 3B). Liver expression of *Id1* mildly (but not significantly) reduced and *Bmp6* was unaffected after 2 weeks of iron deficient diet (Fig 3C and 3D, respectively).

### Effect of dietary iron restriction on susceptibility to murine *M*.*tb* infection

**Fig 4:**
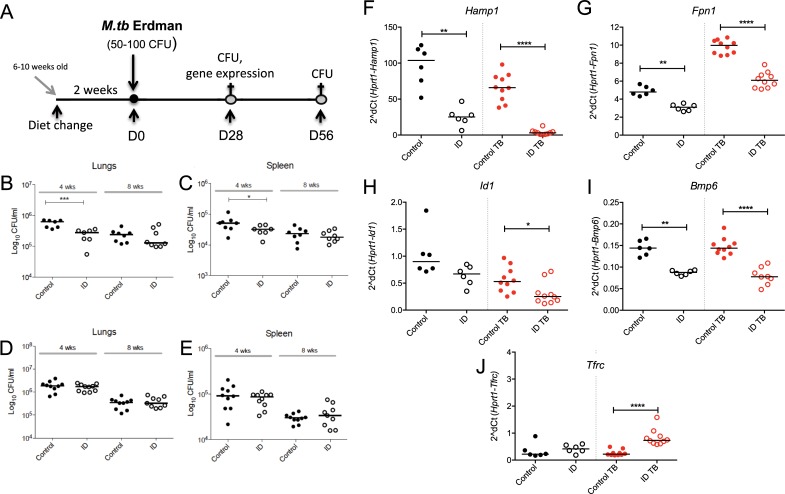
The effect of iron deficiency on susceptibility to *M*.*tb*. Female 6–10 week old C57BL/6 mice were fed an iron deficient (2–6ppm) or control (200ppm) diet for 2 weeks prior to infection with 50–100 CFU of aerosolised *M*.*tb* Erdman (**A**). Mice remained on the respective diet until 4 weeks or 8 weeks post-infection, when animals were sacrificed. Lungs and spleen were harvested for enumeration of CFU (**B-E**) and livers (from 4-week infected animals) for gene expression analyses. Gene expression data is shown for *Hamp1* (**F**), *Fpn1* (**G**), *Id1* (**H**), *Bmp6* (**I**) and *Tfrc* (**J**). Mann-Whitney tests were performed to compare groups where *, **, *** and **** indicate p = <0.05, p = <0.01, p = <0.001 and p = <0.0001, respectively. N = 8 per group for **B** and **C**, n = 10 per group for **D** and **E**. Gene expression data is representative of the two experiments where n = 10 for infected groups and 6 for uninfected controls. In Panels F-J, black symbols represent uninfected animals, red symbols infected animals except for CFU graphs where all animals are infected. Closed circles represent control animals and open circles represent iron deficient animals in all panels.

We next wanted to determine whether restricting iron availability might affect susceptibility to *M*.*tb* infection. Female 6–10 week old C57BL/6 mice were fed either the iron deficient or control diet for 2 weeks prior to *M*.*tb* infection and then continued on their respective diets until they were culled 4 weeks, or 8 weeks later (Fig 4A). Mice on the iron deficient diet for 4 weeks had a significantly reduced bacterial load in the both the lungs and spleens compared to mice on the control diet (p = <0.001 and p = <0.05, respectively), but this difference was not maintained after 8 weeks on the diet (Fig 4B and 4C). Having observed that iron deficiency may result in small decreases in bacterial burden, we performed a second infection experiment to attempt to confirm the above findings. However, in the repeat experiment, no significant differences were observed between the two groups at 4 or 8 weeks, suggesting that the degree of iron deficiency achieved by the employed dietary regime does not robustly and reproducibly affect outcome of infection (p = 0.36 and p = 0.29, for 4-week time points in Fig 4D and 4E), respectively. There were no obvious differences besides bacterial burden between the first and second experiments; animals were age and sex-matched, the same strain, and received a similar dose of *M*.*tb*. This was confirmed by hepatic gene expression data was that were comparable between the two experiments and both showed altered hepatic expression of genes involved in iron homeostasis in both infected and uninfected mice. Fig 4F–4J shows a representative example of gene expression data for the animals from the repeat experiment, whose CFU values are shown in Fig 4D and 4E. In the uninfected mice, *Hamp1*, *Fpn1*, and *Bmp6* mRNA were downregulated in the iron deficient mice compared to control mice (p = <0.01 for all, Fig 4F, 4G and 4I). The same was the case in the infected animals (p = <0.0001, p = <0.0001, p = <0.0001 for *Hamp1*, *Fpn1 and Bmp6*, respectively). *Id1* was also down-regulated in the iron deficient infected mice and *Tfrc* mRNA was up-regulated, when compared to infected mice on the control diet (p = <0.05 and p = <0.0001, respectively, Fig 4I and 4J).

### Effect of more severe iron restriction on body iron parameters

**Fig 5:**
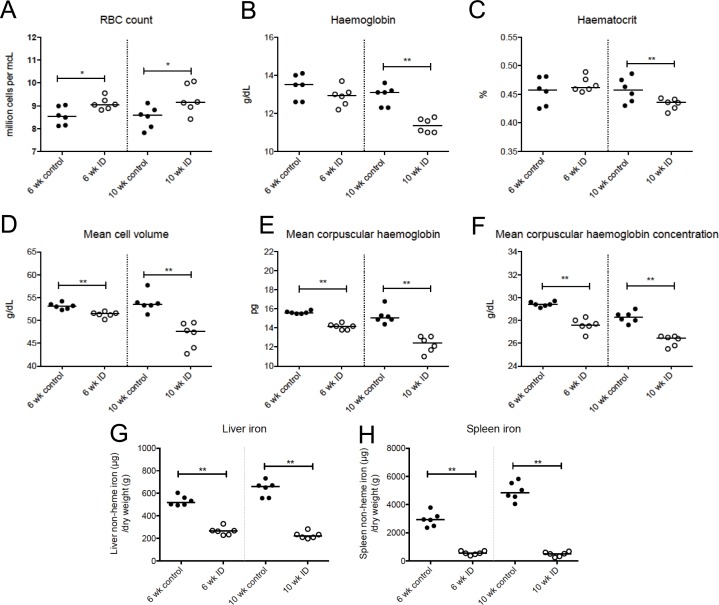
Characterisation of more severe iron deficiency in younger mice. Female 4–5 week old mice were fed an iron deficient (2–6ppm iron) or control (200ppm iron) diet for a total of 6 or 10 weeks. Animals were bled via cardiac puncture under terminal anaesthesia and liver and spleen were removed for quantification of tissue iron. Haematological parameters are shown in panels A-F, and liver and spleen non-heme iron content shown in G-H, respectively. Mann-Whitney tests were performed to compare groups where *, **, *** and **** indicate p = <0.05, p = <0.01, p = <0.001 and p = <0.0001, respectively. N = 6 per group.

Since older mice are likely to have accumulated some iron reserves before being transferred onto a low iron diet, and a low iron diet might therefore take more time to induce iron deficiency, we induced a more severe iron deficiency by feeding younger mice an iron deficient diet for longer prior to infection. We planned to feed female C57BL/6 mice aged 4–5 weeks the iron deficient diet for a total of 10 weeks, consisting of 6 weeks prior to infection with *M*.*tb* and a further 4 weeks of the iron restricted diet post-infection. First, to determine the effects of low iron diet at these timepoints, we analysed iron and haematological parameters in uninfected mice after 6 and 10 weeks of iron restriction. Total RBC counts were increased by low iron diets at 6 and 10 weeks (Fig 5A, p = <0.05), while haemoglobin and haematocrit were significantly reduced only at 10 weeks, indicative of iron deficiency anaemia at this timepoint (p = <0.01 for both, Fig 5B and 5C). Mean cell volume, mean corpuscular haemoglobin, and mean corpuscular haemoglobin concentration were significantly reduced in the mice fed the iron deficient diet when compared with those fed the control diet at 6 weeks and 10 weeks, with stronger effects at the later timepoint(p = <0.01 for all, Fig 5D–5F). In addition, both liver and spleen non-haem iron content was significantly reduced after 6 weeks and 10 weeks on the diet (p = <0.01 in all cases, Fig 5G and 5H). Animals fed the control 200ppm iron diet for 10 weeks total also had higher liver and spleen iron than animals fed the same diet for 6 weeks. Overall, these experiments confirmed that low iron diet for a total of 6 weeks leads to iron deficiency and for 10 weeks causes a more severe iron deficiency and anaemia.

### Effect of more severe iron restriction on susceptibility to *M*.*tb*

**Fig 6:**
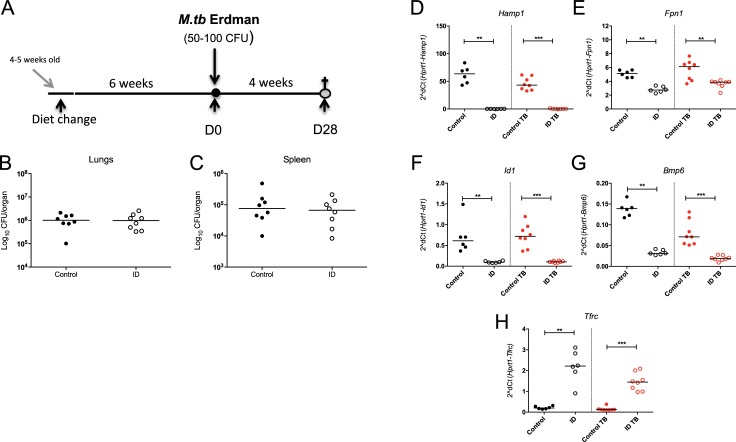
The effect of more severe iron deficiency on susceptibility to *M*.*tb*. Female 4–5 week old C57BL/6 mice were fed an iron deficient (2–6ppm) or control (200ppm) diet for 6 weeks prior to infection with 50–100 CFU of aerosolised *M*.*tb* Erdman (A). Mice remained on the respective diet until 4 weeks post-infection, when animals were sacrificed (a total of 10 weeks on their respective diets). Lungs and spleen were harvested for enumeration of CFU (B+C), and livers for gene expression analyses for *Hamp1* (D), *Fpn1* (E), *Id1* (F), *Bmp6* (G) and *Tfrc* (H). Mann-Whitney tests were performed to compare groups where *, **, *** and **** indicate p = <0.05, p = <0.01, p = <0.001 and p = <0.0001, respectively. N = 8 animals in infected groups and n = 6 in uninfected groups. Black symbols represent uninfected animals, red points infected animals except for CFU graphs where all animals are infected. Closed circles represent control animals and open circles, iron deficient animals.

Next, we wanted to determine whether the severe iron restriction could affect susceptibility to *M*.*tb*. To investigate this, 4–5 week old female C57BL/6 mice were again fed the iron deficient or control diet for a total of 6 weeks before infection with *M*.*tb*, and were culled 4 weeks post-infection (a total of 10 weeks on their respective diets) (Fig 6A). No differences were observed in bacterial burdens in lungs or spleens of animals fed the iron deficient diet, when compared with controls (p = 0.90 and p = 0.72, respectively, Fig 6B and 6C). In the uninfected controls, hepatic levels of *Hamp1*, *Fpn1*, *Id1* and *Bmp6* mRNA were significantly reduced in the iron deficient animals in comparison to those on the control diet (p = <0.01, p = <0.01, p = <0.01 and p = <0.001, respectively, Fig 6D–6G). The same was the case in the *M*.*tb* infected animals (p = <0.001, p = <0.01, p = <0.001, p = <0.001, respectively, Fig 6D–6G. *Tfrc* mRNA was significantly raised in the iron deficient animals, to levels higher than we had previously observed in animals kept on iron deficient diets for shorter time periods (p = <0.01 uninfected animals, p = <0.001 infected animals, Fig 6H). These data indicate that these animals were likely more iron deficient than those in the previous experiments but that nevertheless the severe iron deficiency in this model did not affect bacterial burden.

## Discussion

There is increasing evidence for a role of hepcidin and host iron status in mycobacterial infection [23, 25, 27]. In this study, we used a stringent aerosol *M*.*tb* challenge model to show that hepcidin expression fluctuates following *M*.*tb* infection and is down-regulated at later time points. We also show that neither hepcidin knockout nor dietary iron restriction affect susceptibility to infection.

In the initial time course experiments, we found that *Hamp1* mRNA expression fluctuated post-infection and was significantly decreased at 4 and 8 weeks post-infection in comparison with uninfected controls, contrasting with previous *in vitro* macrophage studies in which *M*.*tb* or *M*.*tb* components stimulated hepcidin mRNA production in a range of cell types [32]. In addition, this also contrasts with evidence that a wide range of infectious stimuli, including the macrophage-tropic bacterium *Salmonella typhimurium*, strongly induce hepcidin transcription [13, 16]. Moreover, the lack of hepcidin upregulation was unexpected since *M*.*tb* infection induces expression of pro-inflammatory cytokines such as tumour necrosis factor alpha (TNF-α), interferon gamma (IFN-γ), and interleukin-6 (IL-6), a strong inducer of hepcidin expression [13, 31, 33]. It is possible that the levels of inflammation and IL-6 induced during the relatively slow-growing *M*.*tb* infection model are not sufficient enough to promote hepcidin induction. It is also possible that an intra-macrophage infection with mycobacteria affects host systemic iron homeostasis in a unique way as there is increasing evidence that iron availability is differentially regulated depending on where the pathogen resides in the host [34, 35]. Moreover, a study of chronic murine infection with *Mycobacterium avium*, which also did not find any evidence of hepcidin up-regulation, supports this concept [36].

We also found that hepcidin mRNA correlated significantly with *Id1*, a marker of BMP-SMAD signalling, and not with *Fga*, a marker of IL-6/inflammatory pathway induction, suggesting that in *M*.*tb*, hepcidin may be regulated by BMP/SMAD signalling rather than by inflammatory pathways, but further work is needed to confirm this. As well as the observed down-regulation of hepcidin mRNA, we also found liver *Fpn1* mRNA to be up-regulated post-infection. Again the direction of this change in expression is counter to that observed in many other inflammatory situations, in which *Fpn1* mRNA expression is down-regulated (reviewed in [37]). There is some evidence that alveolar macrophages express ferroportin and are able to respond to hepcidin [38]. Therefore, we postulated that the observed increase in ferroportin may result in iron release from *M*.*tb* infected macrophages, and may therefore be a protective mechanism—assuming that *Fpn1* mRNA reflects ferroportin protein levels in this case, which is plausible as hepcidin levels are decreased. Indeed, a transient suppression of *M*.*tb* growth has been observed in experiments overexpressing ferroportin in *M*.*tb* infected macrophages [39]. Similarly, a number of *in vitro* studies suggest that the host response to the intracellular pathogen, *Salmonella*, is to elevate ferroportin, thereby increasing iron efflux from the macrophage and reducing bacterial growth [34, 40, 41]–although not all studies agree on this point [42].

Since we found *Hamp1* expression to be down-regulated and *Fpn1* mRNA increased following *M*.*tb* infection, which suggested a host protective response, we investigated whether deletion of the *Hamp1* gene in a mouse model might reduce susceptibility to *M*.*tb* infection. *Hamp1*^*-/-*^ mice have an iron overloaded phenotype similar to that observed in severe forms of hereditary haemochromotosis, including raised serum iron parameters, excess iron accumulation in the liver, and reduced iron in the spleen, as has been well characterised previously [29]. It is important to note that in *Hamp1*^*-/-*^ mice, splenic macrophages and Kupffer cells do not store iron and are relatively iron-spared but likely have a high iron turnover due to inhibited ferroportin activity, whereas other cell types are generally iron overloaded. Pulmonary iron also increases in *Hamp1*^*-/-*^ mice, with iron loading observed in epithelial cells and alveolar macrophages, despite higher expression of ferroportin in the latter [43]. The iron overloading in the lung is likely not due to a lack of lung-produced hepcidin, or hepcidin that can be produced by myeloid cells[44] but because of liver hepcidin deficiency as a hepatocyte-specific hepcidin knock-out recapitulated the pulmonary iron overload seen in the total knockout mouse.

Interestingly, despite iron overload, we found that susceptibility to *M*.*tb* infection did not differ between *Hamp1*^*-/-*^ mice and wild type controls after 28 days of infection, consistent with recent findings by Stefanova *et al*. [28]. This is a stark contrast to the remarkably increased susceptibility of this mouse model to blood borne pathogens such as *Vibrio vulnificus* [1], suggesting that hepcidin plays a vital role in protection against extracellular bacteria by keeping serum iron levels down, but does not play a critical role to protect against *M*.*tb* infection. Humans with HFE-linked haemochromatosis (caused by a relative insufficiency of hepcidin) are also susceptible to severe *V*. *vulnificus*[45], but not it seems, to tuberculosis[3], although definitive evidence is lacking. It is possible that the high turnover of macrophage iron in hepcidin deficient mice may facilitate access of the intracellular bacteria to iron. Treatment of mice with a synthetic hepcidin agonist such as PR73 results in locking of iron inside macrophages [46], and would represent another interesting model to evaluate with regards to *M*.*tb* infection. However, injection of iron-dextran, which leads to macrophage iron accumulation, also did not enhance *M*.*tb* infection [28].

We also investigated the effect of reducing hepcidin expression, and restricting iron availability, by inducing dietary iron deficiency in our mice. Some previous mouse studies suggest that iron overload can worsen the outcome of mycobacterial infection [27, 47, 48] but little is known about the effects of iron deficiency. Here, we showed that mice fed an iron deficient diet for a period of 2 weeks have reduced *Hamp1*, and longer time periods of iron deficiency significantly reduced liver *Hamp1*, *Id1* and *Bmp6* mRNA expression, as well as other parameters such as tissue iron and haematological parameters that confirm iron deficiency. Increased transferrin receptor mRNA also indicates iron deficiency. Iron deficient mice also had reduced hepatic *Fpn1* mRNA. However, induction of iron deficiency, even severe iron deficiency brought about by keeping mice on low iron diets for 10 weeks, did not reproducibly affect infectious outcome in this *M*.*tb* model. There is evidence that both iron deficiency and iron overload may negatively affect the immune response because iron is an essential cofactor for cellular activities. For example, iron deficiency may have a negative impact on lymphocyte-mediated immune responses, which are important for defence against *M*.*tb* [49–51]. Speculatively, any negative impact iron deficiency had on bacterial growth may simultaneously be counterbalanced by negative impacts on the host immune response, which may overall result in no observable differences in bacterial burden. Our experiments assessed *M*.*tb* burden 28 days after infection, a standard timepoint for evaluating the effect of vaccines (eg BCG) on *M*.*tb* infection, and 56 days after infection. It is possible that longer periods of infection could reveal an effect of iron deficiency on bacterial growth *in vivo*. An additional or alternative theory is that *M*.*tb* is resistant to changes in host iron status because of its wide range of iron acquisition mechanisms [52, 53]. *M*.*tb* may be extremely effective at adjusting to even severe iron restriction via increasing expression of iron-scavenging machinery. For example, analysis of the expression of genes encoding mycobacterial iron storage proteins, as well as those involved in siderophore biosynthesis could indicate whether *M*.*tb* growing in the iron deficient animals is responding to altered host iron status *in vivo*. Inhibiting *M*.*tb* iron acquisition through drug targeting is a proposed approach to combat tuberculosis; however siderophores could also act as immune targets via vaccination so that bacterial growth could be constrained by host immunity. Interestingly, the host non-classical MHC protein CD1a can present a mycobacterial pre-siderophore compound dideoxymycobactin to T cells [54], supporting this idea.

In summary, our data demonstrate that liver hepcidin is down-regulated following *M*.*tb* infection, and may be regulated by BMP/SMAD signalling pathways rather than inflammatory pathways. This contrasts with evidence of hepcidin induction following a wide range of other infections, highlighting that host changes to iron metabolism post-infection are pathogen-specific. In addition, we showed that neither hepcidin deficiency nor iron deficiency had a significant impact on outcome of murine *M*.*tb* infection, again contrasting with other infections in which iron deficiency and hepcidin can play an important role. These data highlight that changes in host iron metabolism brought about by hepcidin deficiency or iron deficiency do not critically influence murine *M*.*tb* infection, and reinforce the general idea that altered iron distribution has a considerably different impact depending on the nature of the invading pathogen.

## Supporting information

S1 Fig Hepatic *Hamp1* mRNA in M.tb infected versus uninfected BALB/c mice. Age-matched female animals were infected with M.tb via aerosol for 28 days or left uninfected before liver *Hamp1* mRNA levels were analysed. Mann-Whitney tests were performed to compare groups where * indicates p = <0.05.
(EPS)

S2 Fig Organ iron loading in hepcidin knock-out mice. Female 4–10 month old *Hamp1*^*-/-*^ mice and wild type controls were bled via cardiac puncture under terminal anaesthesia. Haematological parameters are shown in panels A-H. Perls’ staining of lung, liver and spleen sections at an original magnification of x10 is shown in panel I.
(PDF)

S1 Table Table of TaqMan Gene Expression Assays used in qRT-PCRs. (EPS)
