# Supplementary figures and images for: Hepcidin deficiency and iron deficiency do not alter tuberculosis susceptibility in a murine M.tb infection model
Source: PLoS One. 2018 Jan 11;13(1):e0191038. doi: 10.1371/journal.pone.0191038 (PMC5764373; doi:10.1371/journal.pone.0191038)

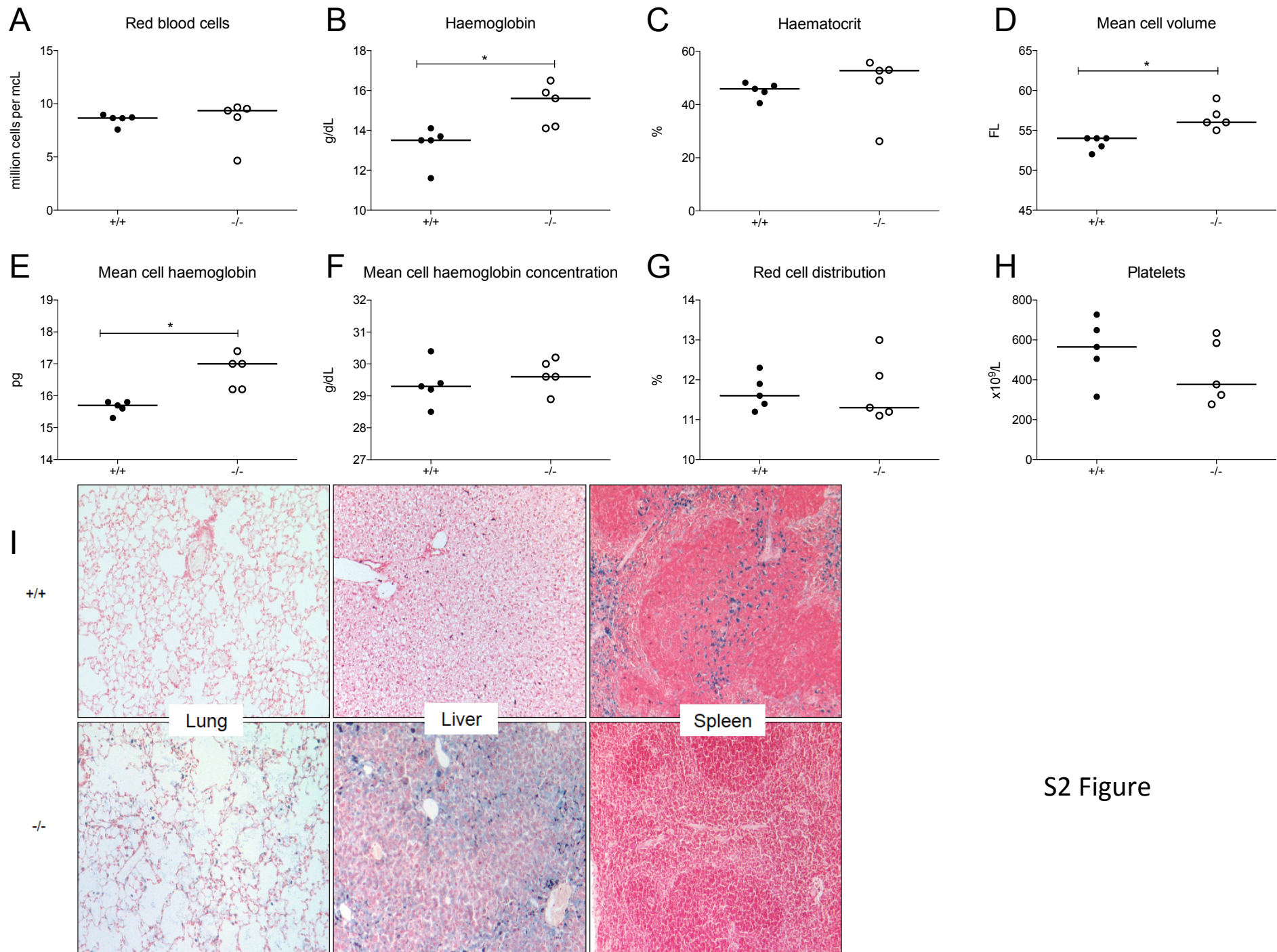

S2 Figure

Supplement: S2 Fig — Female 4–10 month old Hamp1-/- mice and wild type controls were bled via cardiac puncture under terminal anaesthesia. Haematological parameters are shown in panels A-H. Perls’ staining of lung, liver and spleen sections at an original magnification of x10 is shown in panel I. (PDF) [file pone.0191038.s002.pdf]
